# Supplementary material for: An automated pipeline for computation and analysis of functional ventilation and perfusion lung MRI with matrix pencil decomposition: TrueLung
Source: Z Med Phys. 2024 Sep 20;35(4):452–69. doi: 10.1016/j.zemedi.2024.08.001 (PMC12766494; doi:10.1016/j.zemedi.2024.08.001)
Supplement: Supplementary Data 1 [file mmc1.pdf]

Supplementary Material S1 and S2

Supplementary Material S1. *Entire PDF report for one subject with CF.*

Representative full report of pulmonary functions for the same subject with CF presented in Figures 2 and 3. This report includes maps for all the slices acquired.

Functional lung MRI report file

Truelung version: 1.0
Method: MP

Evaluation date: 2022-01-01 15:00
Thresholding: MEDIAN - 75%

Patient data

Name: Family Name, Name
Patient ID: 0123456789
Birth date: 2004-01-01
Age: 17 y/o
Sex: M

Examination data

Station name: MR1
MR scanner: Aera
Baseline: -

Examination date: 2022-01-01 12:00
Sequence: ufssfp
Study ID: Examination TrueLung

Global outcomes

Function	Slices	Volume [mL]	Defects [%]	Mean ± Std [Units]
Ventilation (V)	11	1585	31.3	6.7 ± 3.9
Perfusion (Q)	11	1585	30.9	332.3 ± 176.0

Lobar outcomes

Function	Lobe	Volume [mL]	Defects [%]	Mean ± Std [Units]
Ventilation (V)	LU	369	16.5	8.2 ± 3.4
	LL	372	33.7	6.3 ± 3.7
	RU	305	49.1	5.9 ± 3.8
	RM	164	23.3	7.2 ± 3.1
	RL	374	32.4	6.4 ± 4.3
Perfusion (Q)	LU	369	19.6	353.8 ± 162.1
	LL	372	20.9	366.3 ± 167.6
	RU	305	59.2	240.4 ± 147.1
	RM	164	18.9	461.0 ± 218.2
	RL	374	34.4	310.5 ± 152.5

Ventilation and perfusion maps

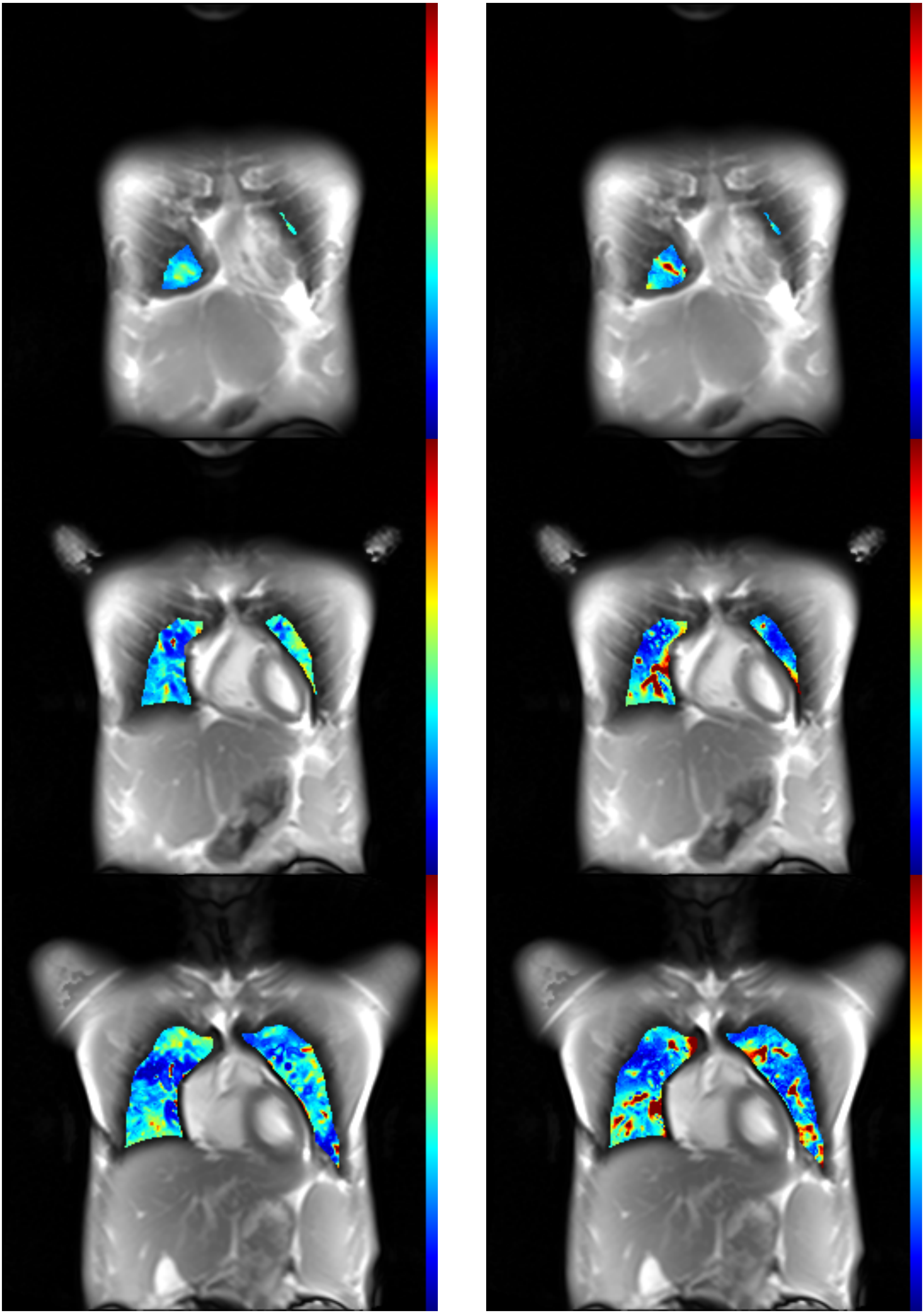

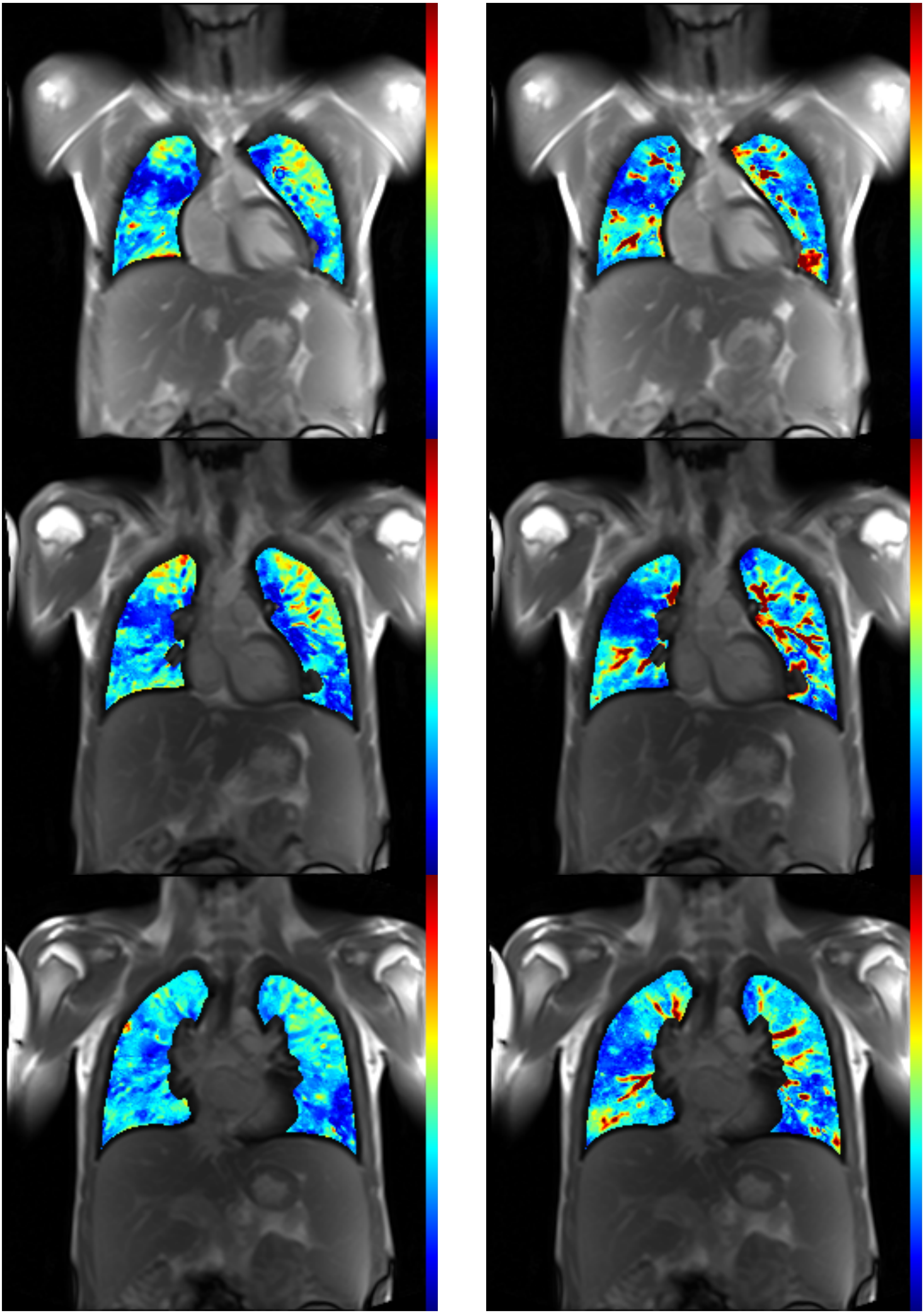

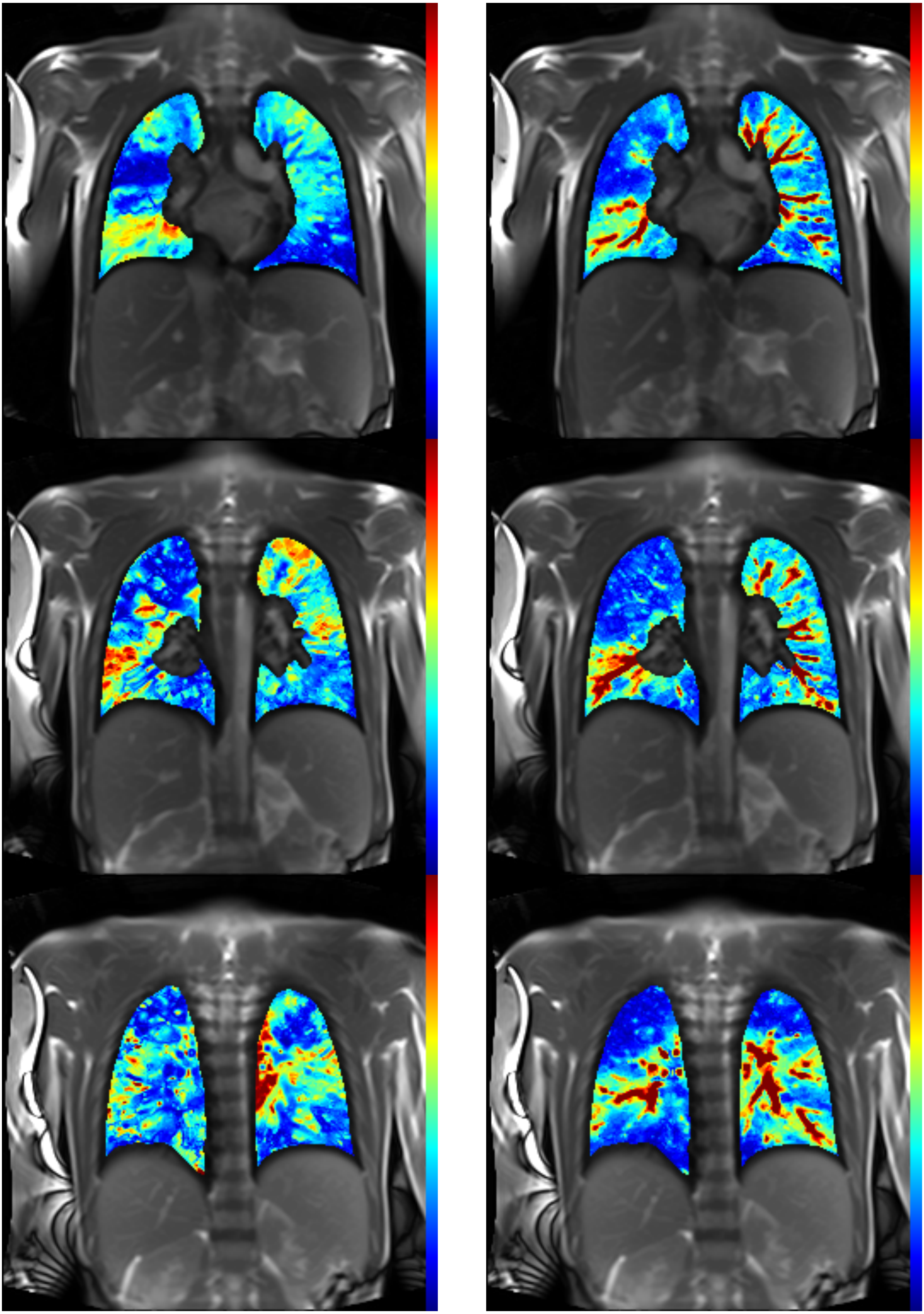

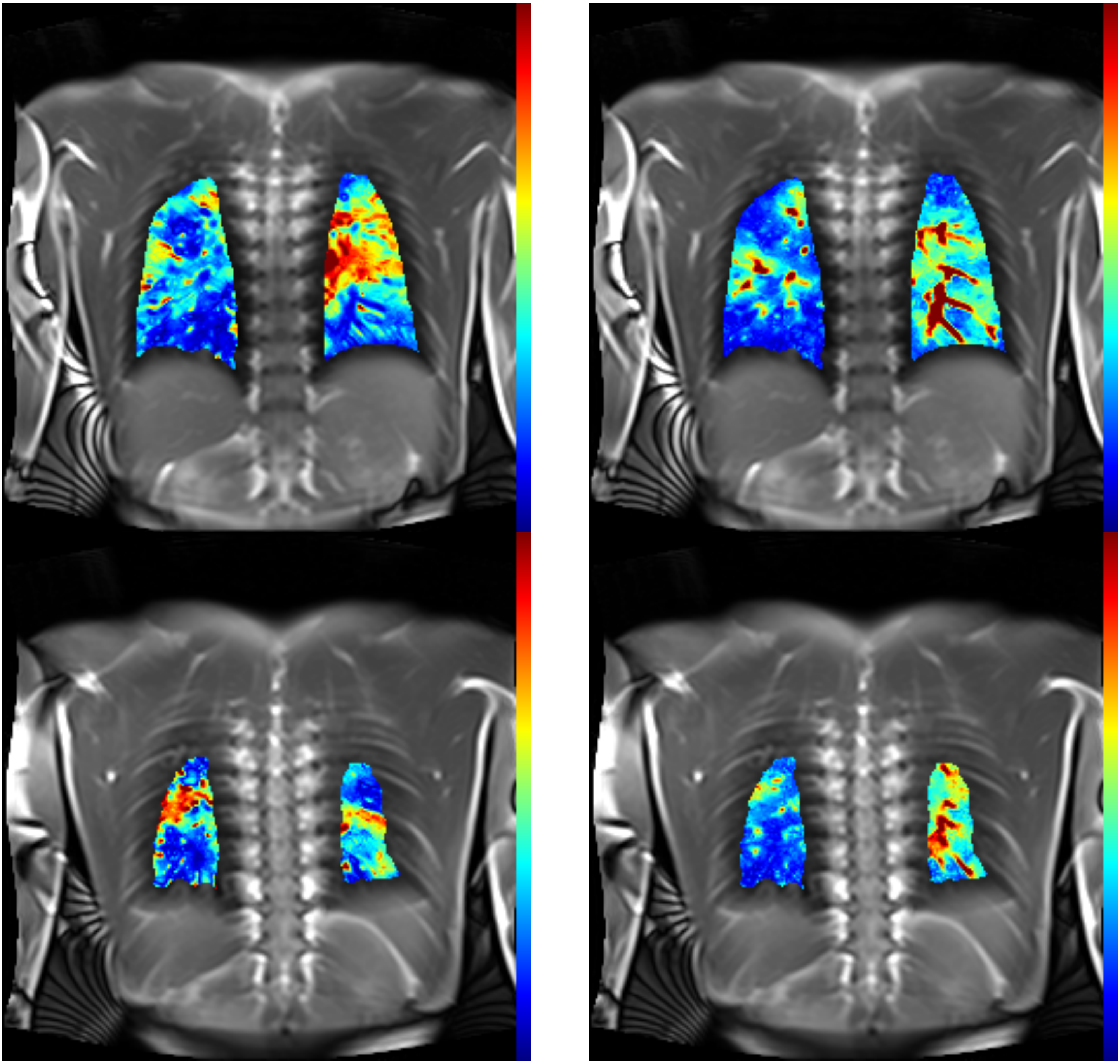

Ventilation and perfusion impairment maps

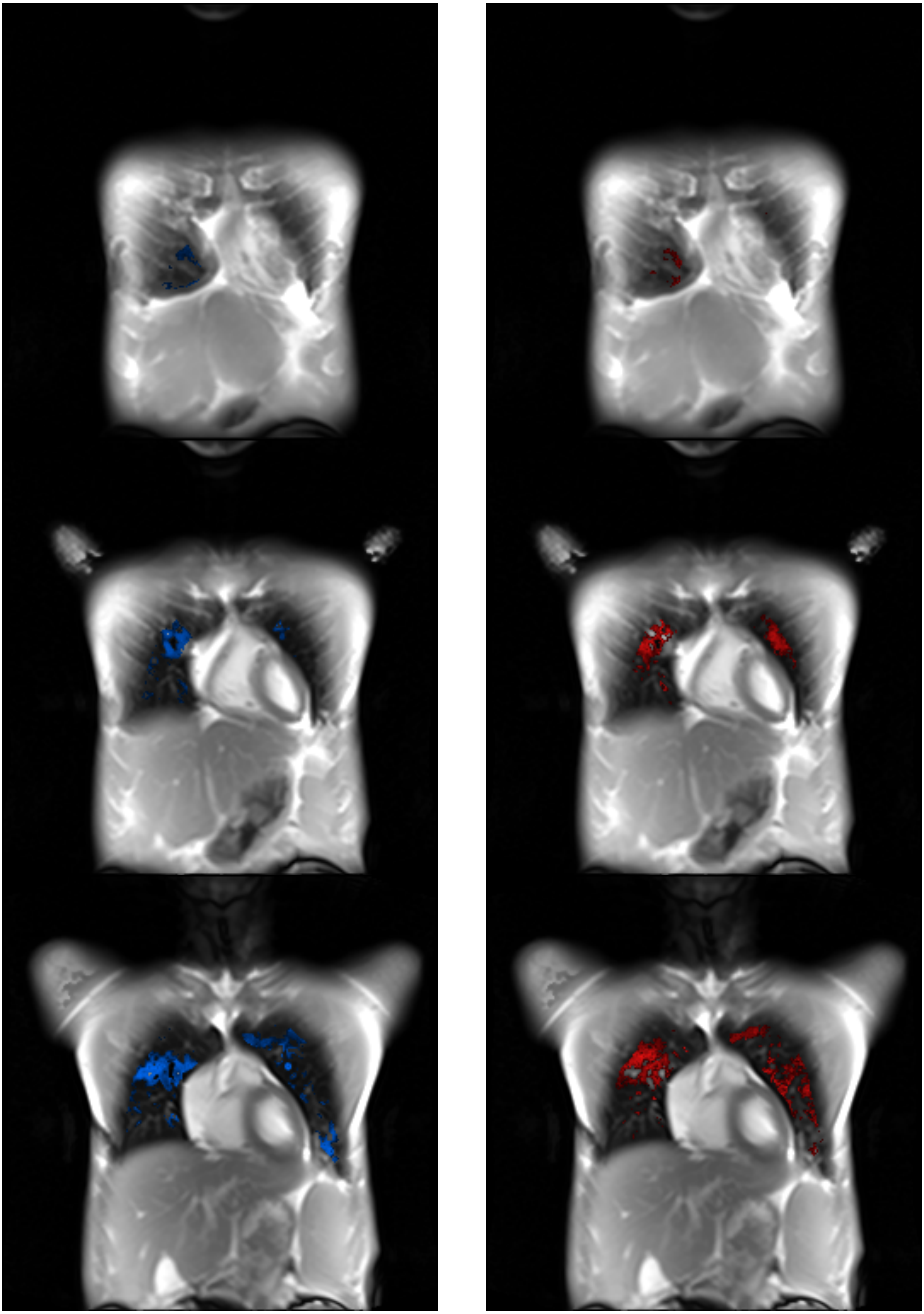

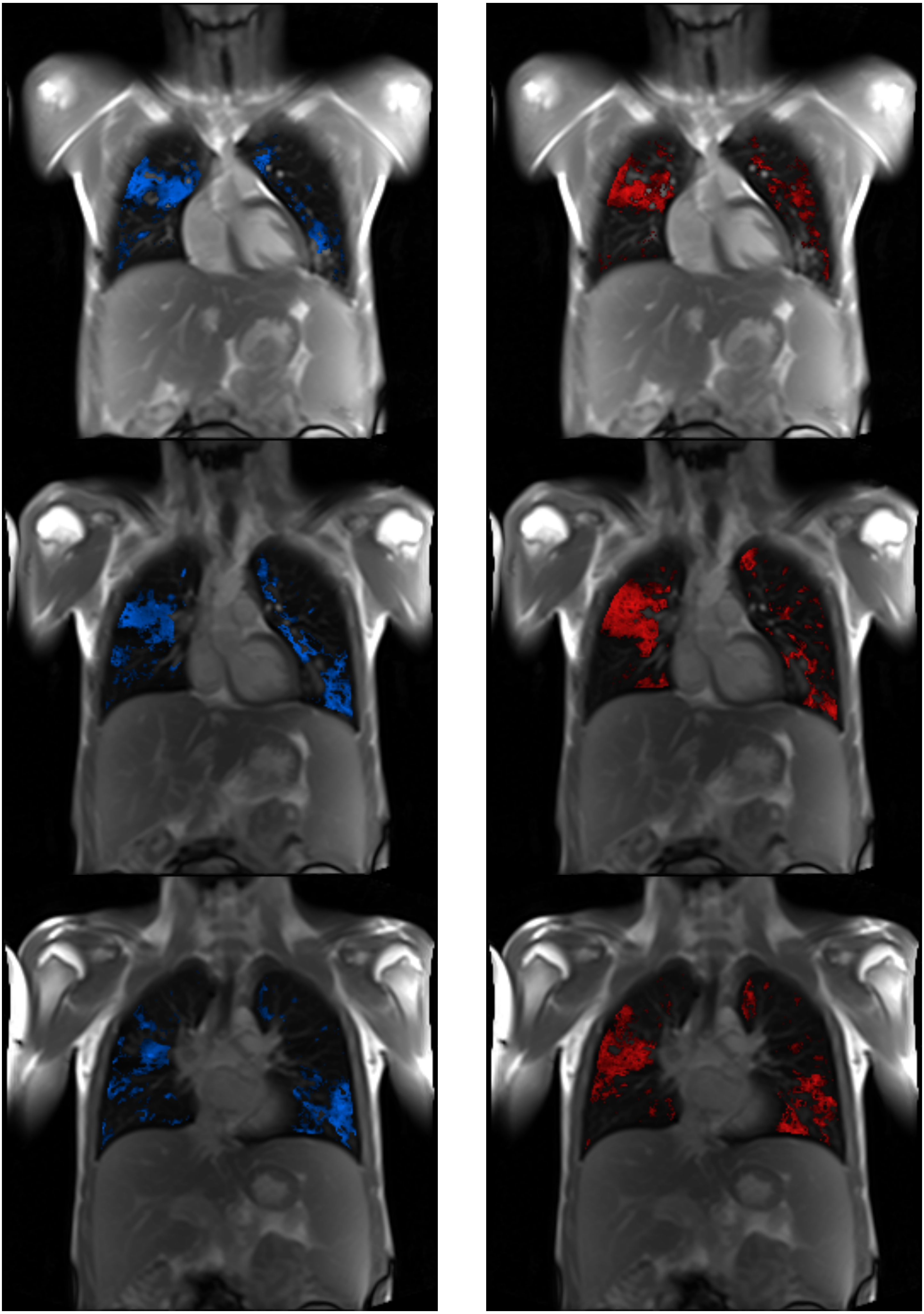

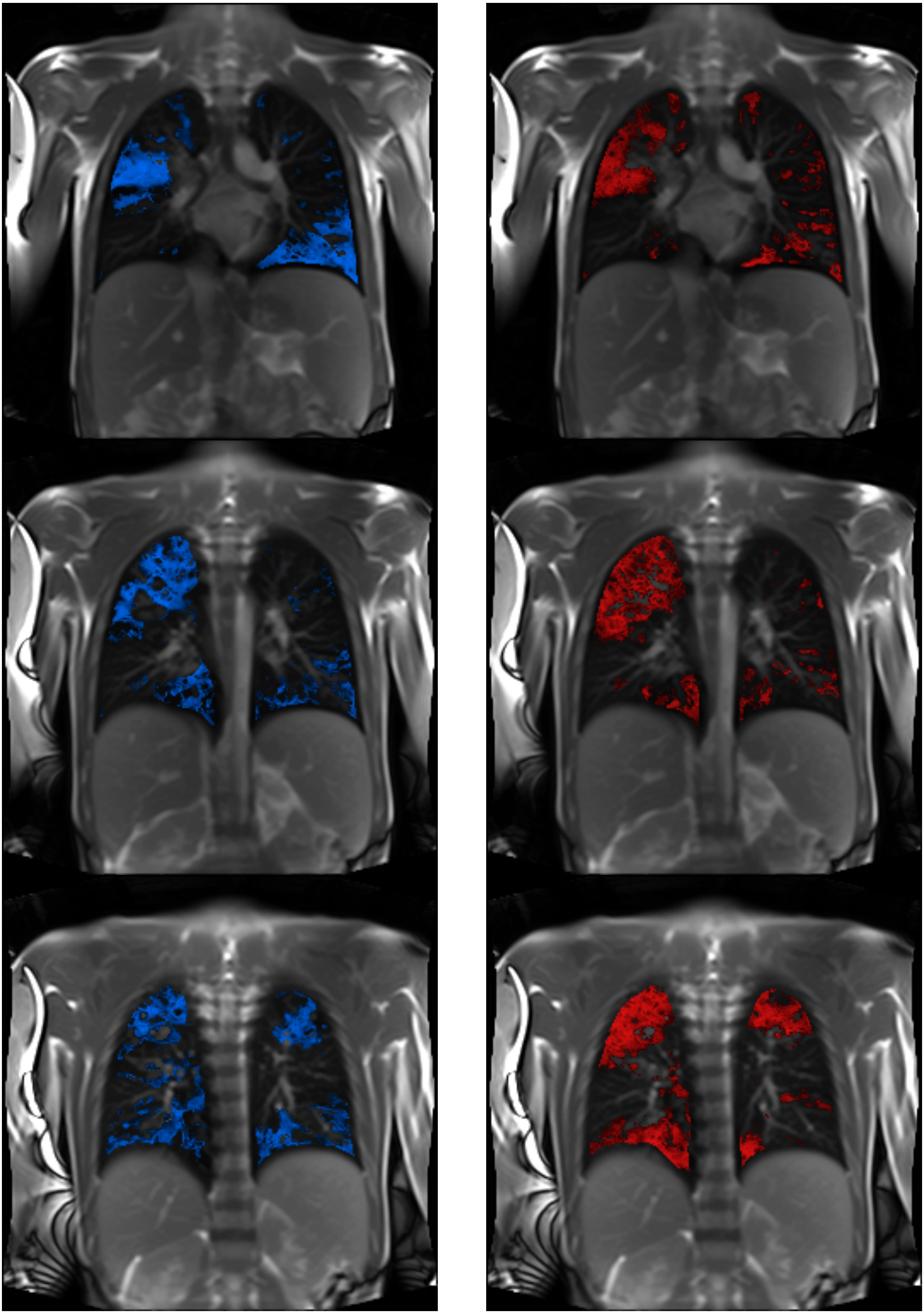

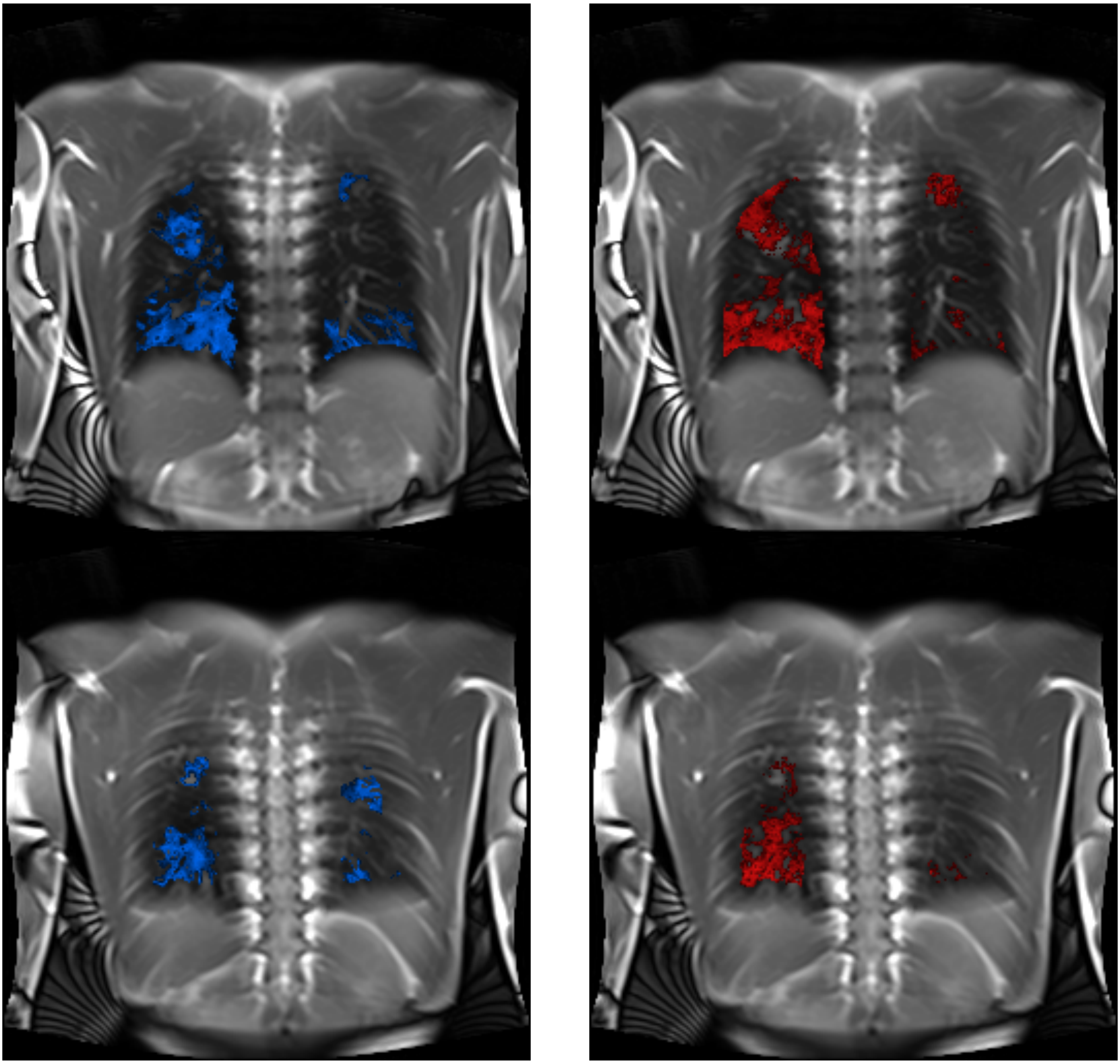

Supplementary Material S2. *Extended tabulated pulmonary data file generated with TrueLung.*

The extended tabulated report-file generated by TrueLung gives information for the whole lung, the lobes, and for every acquired slice. The data for the same subject with CF presented in Figures 2 and 3 as well in the Supplementary Material S1 are given.

Map	N	Volume	Defects	Ratio
V_MAP	11	54143	16918	0.3125
Q_MAP	11	54143	16747	0.3093

Map	N	Volume	Mean	Stddev	Median
V_MAP	11	54143	6.730	3.862	6.229
Q_MAP	11	54143	332.257	176.048	297.476

Map	Lobe	Volume	Defects	Ratio
V_MAP	LU	12614	2080	0.1649
V_MAP	LL	12719	4284	0.3368
V_MAP	RU	10417	5110	0.4905
V_MAP	RM	5618	1308	0.2328
V_MAP	RL	12775	4136	0.3238
Q_MAP	LU	12614	2471	0.1959
Q_MAP	LL	12719	2659	0.2091
Q_MAP	RU	10417	6162	0.5915
Q_MAP	RM	5618	1064	0.1894
Q_MAP	RL	12775	4392	0.3438

Map	Lobe	Volume	Mean	Stddev	Median
V_MAP	LU	12614	8.190	3.410	7.896
V_MAP	LL	12719	6.248	3.728	5.533
V_MAP	RU	10417	5.904	3.781	5.339
V_MAP	RM	5618	7.206	3.056	6.890
V_MAP	RL	12775	6.357	4.326	5.506
Q_MAP	LU	12614	353.831	162.049	327.530
Q_MAP	LL	12719	366.343	167.551	352.786
Q_MAP	RU	10417	240.394	147.069	199.323
Q_MAP	RM	5618	461.016	218.185	450.757
Q_MAP	RL	12775	310.527	152.473	276.781

Map	SL	Area	Defects	Ratio
V_MAP	0	427	102	0.2389
V_MAP	1	1469	363	0.2471
V_MAP	2	3547	1051	0.2963
V_MAP	3	4624	1332	0.2881
V_MAP	4	5475	1664	0.3039
V_MAP	5	6243	1552	0.2486
V_MAP	6	7168	2348	0.3276
V_MAP	7	7773	2519	0.3241
V_MAP	8	8649	2913	0.3368
V_MAP	9	6107	2100	0.3439
V_MAP	10	2661	974	0.3660
Q_MAP	0	427	79	0.1850
Q_MAP	1	1469	486	0.3308
Q_MAP	2	3547	1093	0.3081
Q_MAP	3	4624	1302	0.2816
Q_MAP	4	5475	1621	0.2961
Q_MAP	5	6243	1655	0.2651
Q_MAP	6	7168	2128	0.2969
Q_MAP	7	7773	2508	0.3227
Q_MAP	8	8649	2982	0.3448
Q_MAP	9	6107	2044	0.3347

Q_MAP	10	2661	850	0.3194

Map	SL	Area	Mean	Stddev	Median
V_MAP	0	427	8.338	2.003	8.115
V_MAP	1	1469	9.120	3.789	8.815
V_MAP	2	3547	8.403	3.980	8.319
V_MAP	3	4624	6.314	2.880	6.181
V_MAP	4	5475	4.431	1.946	4.268
V_MAP	5	6243	7.176	2.268	7.241
V_MAP	6	7168	9.684	4.924	9.952
V_MAP	7	7773	6.727	3.697	6.064
V_MAP	8	8649	5.954	3.414	5.395
V_MAP	9	6107	6.233	3.831	5.487
V_MAP	10	2661	3.047	2.050	2.606
Q_MAP	0	427	691.824	165.570	673.343
Q_MAP	1	1469	570.111	263.997	534.357
Q_MAP	2	3547	456.621	192.168	433.435
Q_MAP	3	4624	372.252	142.104	361.807
Q_MAP	4	5475	393.611	168.817	396.510
Q_MAP	5	6243	175.353	56.679	176.583
Q_MAP	6	7168	278.756	110.368	265.053
Q_MAP	7	7773	292.124	125.899	277.977
Q_MAP	8	8649	345.485	190.521	315.711
Q_MAP	9	6107	375.966	165.893	360.217
Q_MAP	10	2661	267.888	121.236	246.010

Method	Evaluation	VoxelSize
mp	median 0.75	29.28

Volume	VolumeDiff	Height	Weight
1585.21	202.34	1.61	54

MeanVFreq	StdVFreq	MeanQFreq	StdQFreq
0.360	0.093	1.256	0.082

Truelung Version: 1.0
